# Preparation and *in vitro* Evaluation of Ethylcellulose Coated Egg Albumin Microspheres of Diltiazem Hydrochloride

**DOI:** 10.4103/0975-1483.62209

**Published:** 2010

**Authors:** TP Shailesh, PD Vipul, JK Girishbhai, CJ Manish

**Affiliations:** *Department of Pharmaceutics and Pharmaceutical Technology, Shri Sarvajanik Pharmacy College, Mehsana - 384 001, Gujarat, India*; 1 *SSR College of Pharmacy, U. T. of Dadra and Nagar, Haveli - 396 230, India*; 2 *L. M. College of Pharmacy, Ahmedabad - 380 009, Gujarat, India*

**Keywords:** Diltiazem hydrochloride, egg albumin microspheres, factorial design, ethylcellulose

## Abstract

The aim of the present investigation was to develop sustained release ethylcellulose-coated egg albumin microspheres of diltiazem hydrochloride (DH) to improve patient compliance. The microspheres were prepared by the w/o emulsion thermal cross-linking method using different proportion of the polymer to drug ratio (1.0:1.0, 1.0:1.5 and 1.0:2.0). A 3^2^ full factorial design was employed to optimize two independent variables, polymer to drug ratio (*X*_1_) and surfactant concentration (*X*_2_) on dependent variables, namely % drug loading, % drug release in 60 min (*Y*_60_) and the time required for 80 % drug release (*t*_80_) were selected as dependable variable. Optimized formulation was compared to its sustained release tablet available in market. The polymer to drug ratio was optimized to 1:1 at which a high drug entrapment efficiency 79.20% ± 0.7% and the geometric mean diameter 47.30 ± 1.5 mm were found. All batches showed a biphasic release pattern; initial burst release effect (55% DH in 1 h) and then were released completely within 6 h. *In situ* coating of optimized egg albumin DH microspheres using 7.5% ethylcellulose significantly reduced the burst effect and provided a slow release of DH for 8-10 h. Finally, it was concluded that ethylcellulose-coated egg albumin DH microspheres is suitable for oral SR devices in the treatment of angina pectoris, cardiac arrhythmias, and hypertension due to their size and release profile.

## INTRODUCTION

Diltiazem hydrochloride (DH) is a calcium channel blocker that relaxes vascular smooth muscle and dilates coronary arteries by altering the calcium flux into the cell.[1] It has been used widely in the treatment of angina pectoris, hypertension, and cardiac arrhythmias.[2] Conventional oral dosage forms of DH are intended to administer dose of 30 mg for four times a day or 60 mg thrice a day. Thus, attempts have been made to develop twice a day of oral sustained release dosage formulation for achieving better patient compliance and relative constant blood levels. It is readily absorbed from the gastrointestinal tract and undergoes substantial first-pass metabolism. The systemic bioavailability of the immediate-release preparation is approximately 40%-50%, with an elimination half-life of 3.5-7 h.[3] Sustained-release DH has been reported to reduce tachycardia without inducing excessive bradycardia and does not significantly reduce baseline heart rate below 74 beats/min.[4] Because of its low bioavailability and short half-life attempts have been made to develop sustained release products and a reduced dosing frequency.[5]

In the last few decades, many types of peroral-modified/controlled release formulations have been developed to decrease dosing frequency and enhance patient compliance, reduce fluctuation in circular drug levels, facilitate a more uniform effect, and improve the clinical efficacy of the drug. These formulations are designed to deliver the drugs at a controlled and predetermined rate, thus maintaining their therapeutically effective concentrations in the systemic circulations for prolonged periods of time.[6,7] There are a few reports on the formulation of oral-controlled release products of diltiazem employing coated beads,[8] pan coating,[9] microencapsulation,[10] and complexation.[11] But due to growing interest in the development of homogeneous monolithic drug delivery system for various routes of administration, a very attractive type of dosage form like ‘microspheres’ is required.

Microspheres are solid approximately spherical particles ranging from 1 to 100 mm in size to provide advantages over sustained release tablets such as ready distribution over a large surface area, predictable and reproducible drug release kinetics, delocalization of total dose in GIT, reduced side effects of drug, and independent drug release rate on gastric transit time.[12,13] Oral multi-unit dosage forms like microspheres have received much attention as modified/controlled drug delivery systems to attribute more uniformly in the gastro-intestine tract, thus resulting more uniform drug absorption and reducing patient-to-patient variability.[14–17] The multi-particulate systems can be filled into hard gelatin capsules or compressed into tablets. It has been reported that the selection of the proper microencapsulation technique and of the excipients, such as polymers as carriers, coaters, and emulsifiers, is very popular in the preparation of the modified release microspheres of the drug.[14–20] In last few years, a number of studies have been made on the compression of microparticles, such as microspheres, microcapsules, and microsponges into tablets, and the effects of excipients and of the compression on the drug release rate from these formulations have been investigated.[18–23] However, not a single study has been done on the filling of the matrix type microspheres of DH as compared to few studies on the tableting of the matrix type microspheres of DH.[24]

Natural biodegradable polymeric-based microspheres as a drug carrier are widely used as they improve the safety and efficacy of drug delivery, drug targeting to specific cells or organs, and better patient compliance. Egg albumin can be used as a carrier for microsphere formulation of drugs as it has properties of protein binding and physical entrapment, the passive and facilitated release process of various types of incorporated drugs from the albumin matrix. To overcome rapid dissolution of highly soluble polymer ‘egg albumin’ in aqueous environment, different cross-linking methods have been used for microsphere preparation. Ethylcellulose, which is one of the most widely used water-insoluble polymers, in modified release dosage forms, was chosen as the matrix-forming polymer due to its good compressibility, excellent physicochemical stability, and minimum toxicity.[15,17]

Hence, the purpose of the present research was to develop microspheres of DH to reduce its dosing frequency using egg albumin as a biodegradable, biocompatible carrier and an ethylcellulose as a coating polymer by the w/o emulsion thermal cross-linking method with a goal of delivering the same dose of the drug through microspheres in a capsule taken once rather than two or three times a day.

## MATERIALS AND METHODS

### Materials

Diltiazem hydrochloride was a gifted from Cadila Pharmaceutical Ltd., Ahmedabad, India. Egg albumin flakes and light paraffin oil of Laboratory Rasayana grade were procured from Ases laboratory, India, and Gujarat Pharmaceuticals Ltd., India, respectively. All other chemicals and solvents used were of analytical grade. The distilled water used was prepared in our laboratory.

### Methods

#### Preparation of egg albumin microspheres

Egg albumin microspheres of DH were prepared by the w/o emulsion thermal cross-linking method with minor modification.[25–29] Hundred ml of light paraffin oil was placed in a glass beaker and mixed with 0.4% w/v span 60 solution by stirring and heating at 70°C for solubilization. The mixture was allowed to cool at room temperature. Add 10 ml of egg albumin aqueous solution of a different drug to polymer ratio ((1.0:1.0, 1.0:1.5 and 1.0:2.0) drop wise (various concentrations 5% w/v, 7.5% w/v, 10% w/v, and 15% w/v) to the different using a 22-gauge hypodermic syringe into an external phase. Light paraffin was stirred at 600 rpm for 10 min with the help of a magnetic stirrer (Remi equipment, 5 l capacity, Mumbai, India). A w/o emulsion was formed. The temperature of the oil bath was raised to 95°C {as preliminary study was carried out at four different temperature levels (30°C, 60°C, 80°C and at 95°C), indicating small, spherical highest percentage yielding microspheres with moderate aggregation at 95°C} and stirring was continued until microspheres were completely dehydrated. Microspheres were then separated by decantation and washed six times with 20 ml of petroleum ether for 2 min at 700 rpm to remove traces of oil. Finally, microspheres were washed three times with 60 zz ml of distilled water for 2 min at 700 rpm and dried at room temperature (at 25°C ± 0.5°C, 60% RH) for 24 h. After drying, a fine yellow free flowing powder was obtained that was stored in desiccators at room temperature. Different batches of microspheres were prepared using different concentrations of surfactant (span 60; 0.2% wt/vol, 0.4% wt/vol, and 0.6% w/v) and different drug to egg albumin ratios (1.0:1.0, 1.0:1.5 and 1.0:2.0). In each case, the other variables were kept constant. Average size of microspheres was determined by using a calibrated stage micrometer. A total of nine batches, each in triplicate, were prepared as per 3^2^ factorial designs.

#### Determination of percentage drug entrapment

Efficiency of drug entrapment for each batch was calculated in terms of percentage drug entrapment (PDE) as per the formula: PDE = 100 (practical drug loading/theoretical drug loading). Theoretical drug loading was determined by calculation assuming that the entire drug present in the egg albumin solution gets entrapped in microspheres and no loss occur at any stage of preparation of microspheres. Practical drug loading was determined by crushing 100 mg of DH loaded egg albumin microspheres in a dry glass mortar containing 100 ml of distilled water. Resultant dispersion was kept aside for 3 h and 1 h in a sonicator. The dispersion was filtered through whatman filter paper (0.22 mm) and the filtrate was analyzed by using a UV/ Vis spectrophotometer (Hitachi U-2000) at 236.5 nm after suitable dilution with distilled water.[30]

#### Determination of mean particle size and particle size distribution (1)

The microspheres were characterized for size distribution by a simple optical microscope (Labomed CX RIII, India), using a calibrated stage micrometer. Geometric mean diameter (GMD) in mm was calculated using the formula: *X_g_* = 10 (*n_i_* log *X_i_*)/*N*) where, *X_g_* is the geometric mean diameter, *n_i_* is the number of particles in the range, *X_i_* is mid-point of the range, and N is the total number of microspheres (*N* = 500).[31]

#### *In vitro* drug release studies

Release of DH from the prepared batches of microspheres were studied in distilled water (900 ml) as prescribed for diltiazem hydrochloride sustained release dosage forms in USP XXIV using apparatus I. To mimic the stomach and intestinal environments conditions, *in vitro* release study[32] was also carried out for the optimized best batches of microspheres using USP dissolution rate test apparatus (Apparatus I, 100 rpm, 37 ± 0.5°C) for the first 2 h in pH 1.2 buffer (900 ml simulated gastric fluid without enzyme) and then dissolution medium was replaced with pH 7.4 phosphate buffer (900 ml) without enzyme and tested for the drug release for another 10 h.[32–34] A muslin cloth (200#) was tied over the basket to prevent slippage of microspheres from the basket during all dissolution studies. Microspheres equivalent to 90 mg of DH in Hard gelatin capsules (transparent, 0 size) were used for dissolution studies. 10 ml of the dissolution sample was withdrawn at different time intervals, filtered through a 0.45 mm membrane filter and replaced with an equal quantity of fresh dissolution medium. The samples were suitably diluted with dissolution fluid and analyzed for DH content spectrophotometrically at 236.5 nm. Percentage drug dissolved at different time intervals was calculated using the Beer’s-Lambert equation (*Y* = 0.05125X + 0.0335, *R*^2^ = 0.997). The time required for 80 % drug release (*t*_80_) was calculated using Korsmeyer and Peppas model.[35,36]

#### Confirmation test for cross-linking

Confirmation of thermal cross-linking of DH loaded egg albumin microspheres was done by solubility and biurate test methods.[37]

#### Optimization usaing factorial design

In this investigation, the drug to polymer ratio and surfactant concentration were selected as independent variables, whereas drug loading was chosen as dependent variables. The other variables such as volume of light paraffin oil (100 ml), volume of aqueous albumin solution (10 m of 7.5% w/v), thermal cross-linking temperature (95°C), thermal cross-linking time (60 min), and stirring speed (600 rpm) were kept constant. The formulation parameters and % yield of the factorial design batches (F1 to F9) are shown in Table 1.

**Table 1:** A 3^2^ full factorial design layout

Batch code	Variable levels in coded forms		Y_60_ (%)	Y_360_ (%)	*t*_8_	*t*_50_	Drug entrapment efficiency (%)	GMD ± SD (µm)
	*X*_1_	*X*_2_						
F1	–1	–1	55.17	78.42	367	54	61.76	52.13 ± 1.1
F2	–1	0	57.00	78.08	369	53	79.2	47.30 ± 1.5
F3	–1	1	56.22	78.73	366	53	65	46.50 ± 2.1
F4	0	–1	51.65	79.16	363	58	59.5	61.40 ± 2.6
F5	0	0	50.45	77.29	37	360	62.25	47.50 ± 1.9
F6	0	1	50.38	78.13	369	60	52.5	47.10 ± 3.1
F7	1	–1	48.96	76.01	379	61	55.5	74.41 ± 2.5
F8	1	0	48.23	76.81	375	62	58.25	66.63 ± 2.8
F9	1	1	47.14	75.42	382	64	48	59.65 ± 1.6
**Translation of coded levels in actual units**								

**Variable level**	**Low (–1)**		**High (+1)**					

Drug to polymer ratio (*X*_1_)	1.0:1.0		1.0:2.0					
Surfactant concentration (*X*_2_)	0.2		0.6					

Y_60_, Y_360_ = the percentage drug released in 60 min and 360 min, *t*_80_ = the time in minutes required for 80% drug dissolution All the batches were prepared using 10 ml 7.5% w/v egg albumin solution, 95°C cross linking temperature, 100 ml light paraffin oil and 60 minutes cross linking time

#### Coating to egg albumin microspheres of diltiazem hydrochloride

To get desired sustained drug release from the microspheres, the burst effect of drug release should be reduced. Hence, egg albumin microspheres of optimized batch F2 (highest drug loading and good sphericity of microspheres) was taken for *in situ* coating using enteric polymer ethylcellulose. Coating solution of ethylcellulose of difference concentration (2.5%, 5.0% and 7.5% w/v) was prepared in acetone with dibutyl phthalate (2% v/v of polymer) as a plasticizer. It was added drop wise to the dispersion containing DH-loaded egg albumin microspheres. The system was stirred for 30 min, filtered (0.45 m size filter medium), washed six times with 6 ml volumes of petroleum ether, and dried at room temperature (25°C ± 0.5°C, 60% RH) for 24 h. A total of three batches, each in triplicates, were prepared to optimize the percentage ethylcellulose coating on batch F2 microspheres.

#### Stability studies

To assess long-term stability,[38] the ethylcellulose-coated egg albumin microsphere formulations in triplicate (batch C3 of 7.5% w/v ethylcellulose coating) were put in hard gelatin capsules (transparent, 0 size) and sealed in aluminum packaging coated inside with polyethylene. The studies were performed at 40°C/75% RH in the stability chamber (Stability Oven, Nirmal Instruments, Delhi, India) for 3 months. At the end of storage period, the formulation was observed for physical appearance, size, shape, surface morphology, drug content, and *in vitro* drug release studies.

#### Comparison of *in vitro* drug release of optimized egg albumin microsphere, batch C_1_ to C_3_ and market formulation

*In vitro* dissolution study in triplicate was carried out for the coated microsphere batches (C_1_, C_2_, and C_3_) and uncoated DH-loaded egg albumin microspheres, using the same method as described earlier in comparison to market formulation of DH microspheres (90 mg Angezem^®^ CD; Capsule: Aztec: Sun Pharmaceutical Research center, Baroda, Gujarat, India).

## RESULTS AND DISCUSSION

### Preliminary trials

The presence of 0.4% w/v span 60 (HLB 5-6) was found to be essential to minimize aggregation of egg albumin droplets during the emulsification step and effective to achieve spherical, small, regular, discrete microspheres with good yield. The type and quality of the oil phase played an important role in controlling the diameter of microspheres. Two types of liquid paraffin (light or heavy) and cottonseed oil were tried. When cottonseed oil and heavy liquid paraffin were used, greater resistance was offered in the process of dispersion, leading to the formation of aggregated irregular-shaped microspheres compared to that of light liquid paraffin. Thus, light liquid paraffin oil was selected and effect of its volume (75, 100, and 150 ml) on the characteristics of microspheres was studied. Discrete, small, regular, and spherical microspheres were obtained when 100 ml of light paraffin oil was used. To study the effect of stirring speed, microspheres were prepared at 100, 250, and 600 rpm. Microspheres with good sphericity were obtained at 600 rpm.

The geometric mean diameter of egg albumin microspheres varied from 47.30 ± 1.5 µm to 59.65 ± 1.6 µm with varying egg albumin concentration. The average percentage drug entrapment was found to be 69.6 ± 4% in all the microsphere formulations. The highest drug loading efficiency was found with 7.5% w/v egg albumin. Three different ratios of drug to egg albumin (1.0:1.0, 1.0:1.5 and 1.0:2.0 wt/wt) were tried. Microspheres prepared using the 1.0:1.0 wt/wt drug to egg albumin ratio showed highest *t*_80_ (389 min), % drug entrapment efficiency (79.20%), and small (GMD: 47.30 ± 1.5), spherical, discrete microspheres. A higher concentration of egg albumin produced a more viscous dispersion, which formed larger droplets and consequently larger microspheres as reported by Pongpaibul *et al*.[39] However, the microspheres showed a burst effect (55% drug release in 1 h) and a relatively faster drug release thereafter (total drug release in 6 hour). *In situ* coating of microspheres with ethylcellulose significantly reduced the burst effect (5% drug release in 1 h).

### Effect of emulsifier concentration on characteristics of microspheres

The geometric mean diameter of microspheres was found to vary from 52.13 ± 1.1 µm to 46.50 ± 2.1 µm on varying emulsifier (Span 60) concentration. Increased surfactant concentration led to the formation of microspheres with a lower GMD as reported by Chemtob *et al*.[37] Increasing Span 60 concentration from 0.2% to 0.6% wt/vol led to stabilization of the emulsion droplets avoiding their coalescence, resulting in smaller microspheres.[40,41] The drug loading efficiency varied from 60.70 ± 0.9% to 78.13 ± 1.3% with varying emulsifier concentration from 0.2% to 0.6% during preparation of egg albumin microspheres.

The GMD of egg albumin microspheres decreased from 61.71 ± 2.0 µm to 47.30 ± 1.5 µm with increasing speed of the magnetic stirrer from 100 rpm to 600 rpm. This result was expected because high stirring rates provide the shearing force needed to separate the oil phase into smaller globules. The stirring speed of 600 rpm was found to be optimum for egg albumin microspheres, as the drug loading efficiency was 78.21 ± 1.6% at this speed. High stirring speed produced an irregular shape of microspheres, but an increased drug entrapment efficacy was found. Stirring time of 60 min was found to be optimum for egg albumin microspheres because at this time period, the microsphere size was small with good drug loading efficiency of 70.25 ± 1.2%.

### Effect of cross-linking temperature and time on microspheres

An increase in cross-linking temperature from 60°C to 95°C leads to a decrease in GMD. This is due to increase in the degree of congealing or rigidization of egg albumin[42] that ultimately results in shrinking of the particles irrespective of the cross-linking time. Increase cross-linking time from 60 min to 120 min irrespective to cross-linking temperature did not show any significant change in GMD. At 95°C cross-linking temperature, cross-linking time of 60 min was enough for conversion of water from liquid state to vapor and hence complete removal of water present in the dispersed phase. This process starts essentially when the time of cross-linking is nearly 50 min, indicating no need to increase cross-linking temperature to get discrete spherical small with high percentage drug loading microspheres. Further increase in cross-linking time at the same temperature did not give the desired product characteristics but showed a step fall in the drug entrapment efficiency; it may be due to the loss of the drug present on the surface of the microspheres to the dispersion medium. It is surface-bound drug that is loosely held and usually lost to the externally oily phase during preparation or to solvents during the washing stage.[43] Increase in cross-linking time at constant cross-linking temperature did not have a significant effect on the % drug loading.

### Full factorial design

The results depicted in Table 1 clearly indicate that the selected dependent variables are strongly dependent on the selected independent variables as they show a wide variation among the nine batches (F1 to F9). The main effects (*X*_1_ and *X*_2_) represent the average result of changing one factor at a time from its low to high value. The interactions (*X*_1_ *X*_2_) show how the response value changes when the two factors are simultaneously changed. The polynomial terms (*X*_1_ *X*_1_ and *X*_2_ *X*_2_) were included in the model to investigate non-linearity. The fitted equations (full models) relating the response, i.e. the %drug loading efficiency, to the transformed factor are shown in Table 1. The polynomial equations can be used to draw conclusions after considering the magnitude of coefficient and the mathematical sign it carries, i.e. positive or negative. The high value of correlation coefficient [Table 1] for the dependent variable indicates a good fit. The equation may be used to obtain estimates of the response since small error of variance was noticed in the replicates.

The polynomial equations relating the responses *Y*_60_ and *t*_80_ to the transferred factors are:

$$Y_{60}\;=\;51.0311\;–\;4.01X_{1}\;–0.34X_{2}\;–0.7175X_{1}\;X_{2}\;+\;1.2933X_{1}X_{1}–0.3067X_{2}X_{2}$$

(2) $$\left(r\;=\;0.9930,\;\mathrm{DF}\;=\;8,\;F\;=\;42.74\right)$$

(3) $$t_{80}\;=\;369.22\;+\;5.66X_{1}\;+\;1.333X_{2}\;+\;1.00X_{1}X_{2}\;+\;4.667X_{1}X_{1}\;–\;1.3333X_{2}X_{2}$$

(3) $$\left(r\;=\;0.8970,\;\mathrm{DF}\;=\;8,\;F\;=\;2.47\right)$$

**Figure 1:**
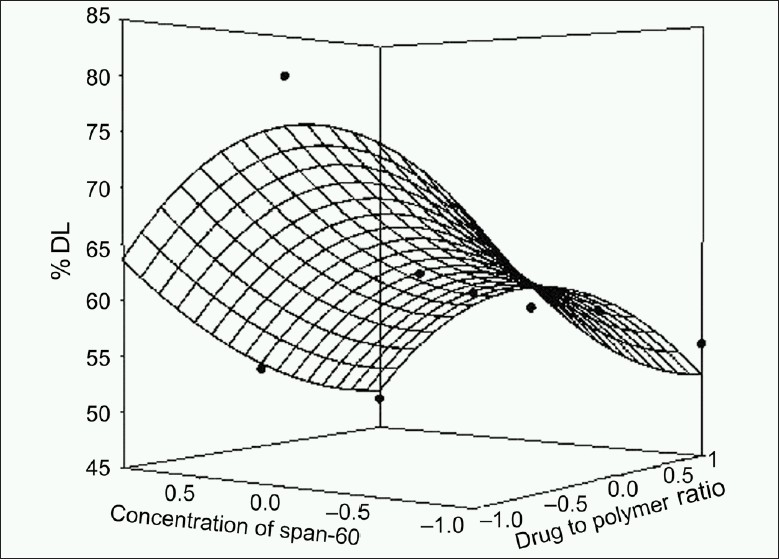
Response surface plot for % drug loading

**Figure 2:**
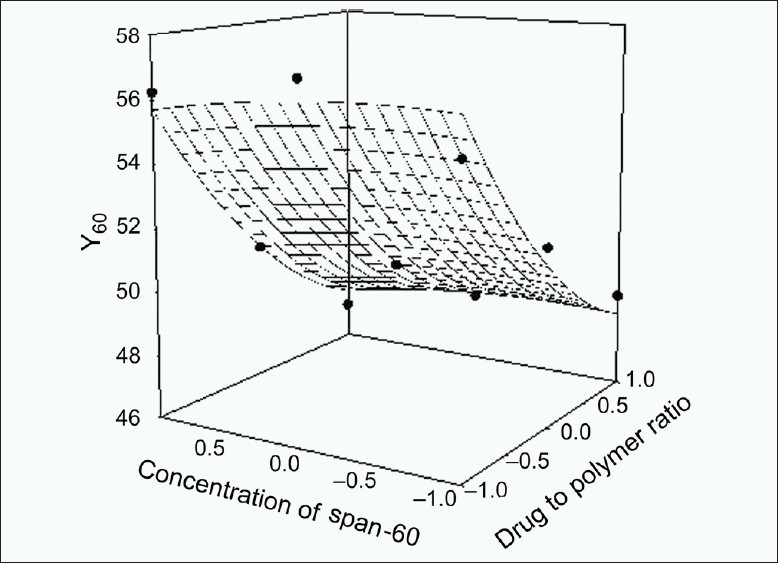
Response surface plot for Y_60_

**Figure 3:**
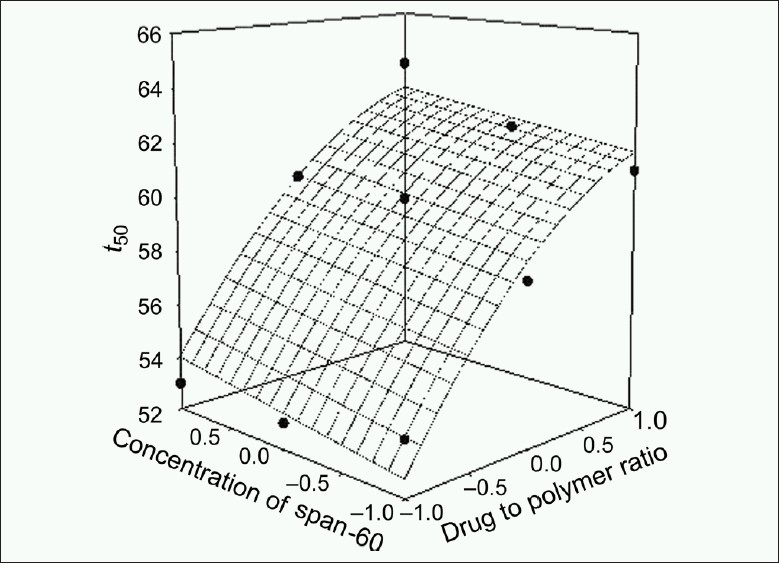
Response surface plot for *t*_50_

**Figure 4:**
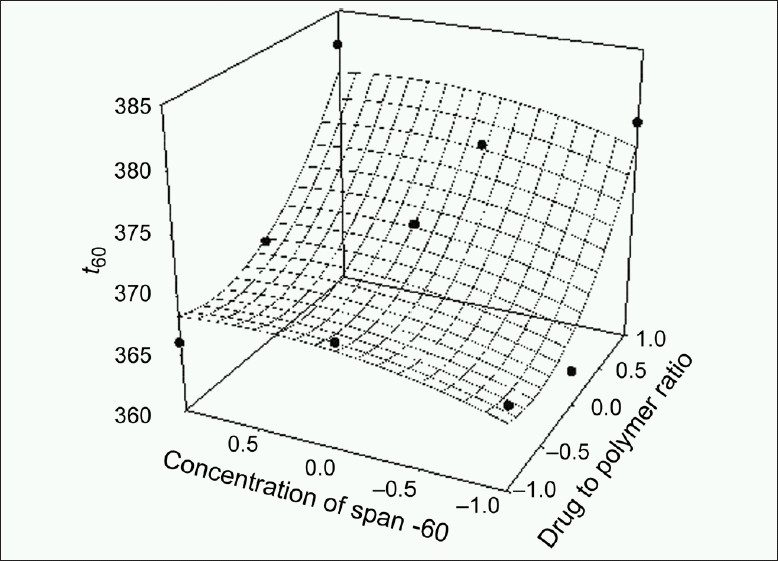
Response surface plot for *t*_80_

The response values for the nine batches showed a wide variation; *Y*_60_ ranged from 47.14 to 57.00% and *t*_80_ ranged from 291 to 395 min. The data clearly indicate that *Y*_60_ and *t*_80_ values depend significantly on the selected independent variables. The high value of correlation coefficient (*r* = 0.8970 for *t*_80_ and *r* = 0.9930 for *Y*_60_) indicates a good fit. The equation (2) represents that the coefficients associated with *X*_1_ carry a negative sign. The *Y*_60_ was found to be inversely related to the polymer to drug ratio. Figures 1–4 show the response surface plots drawn using Sigma Plot software (Jandel Scientific Software, San Rafael, CA).

### Kinetic modeling of drug release

The method of Bamba *et al*. was adopted to decide the most appropriate model.[44] For the optimize batch (Batch F2), the release profile fitted to the Korsmeyer and Peppas model (F = 4.41) giving the least residual sum of squares as compared to the Weibull model (F = 6.03) and Higuchi model (F = 98.83). This superiority is, however, statistically insignificant as shown by the F-ratio test. For the batch F_2_, the values of correlation coefficient were found to be 0.97, 0.97, and 0.94 for Korsmeyer and Peppas, Weibull, and Higuchi models, respectively. Out of nine batches, the Korsmeyer and Peppas model fitted well to all batches except one. Thus, the Korsmeyer and Peppas model was selected. For the Korsmeyer and Peppas model, the values of slope and intercept were found to be 0.5219 and –0.7548, respectively. From the value of slope (*n* = 0.5219, 0.45 < *n* < 0.89), it can be concluded that the drug is released by diffusion of anomalous type (non-Fickian).

### Selection of best batch

**Figure 5:**
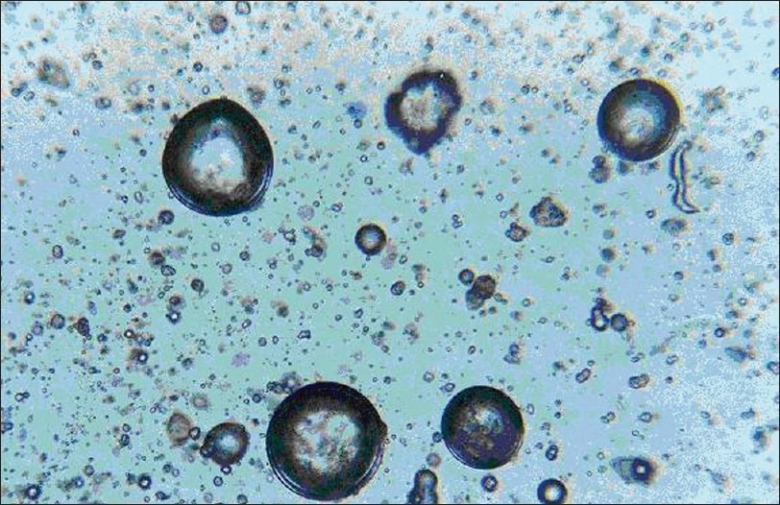
Photomicrographs of DH loaded egg albumin microspheres of optimized batch F2 during process (original magnification ×100)

**Figure 6:**
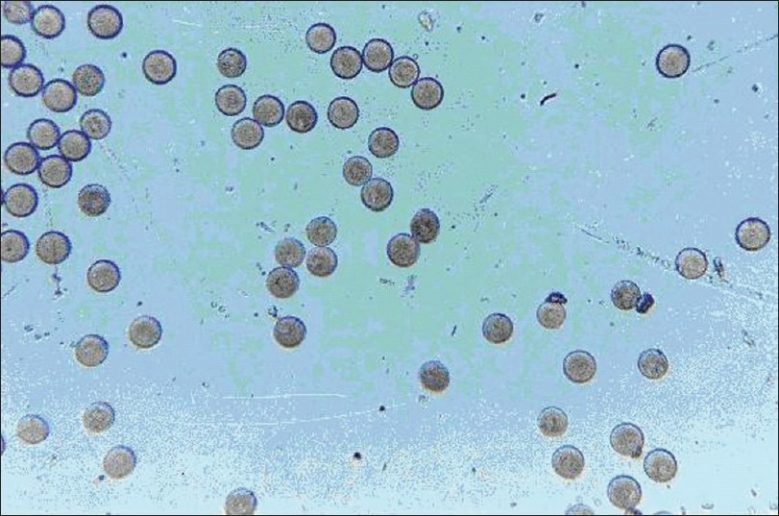
Photomicrograph of dry DH-loaded egg albumin microspheres of batch F2 (original magnification ×100)

**Figure 7:**
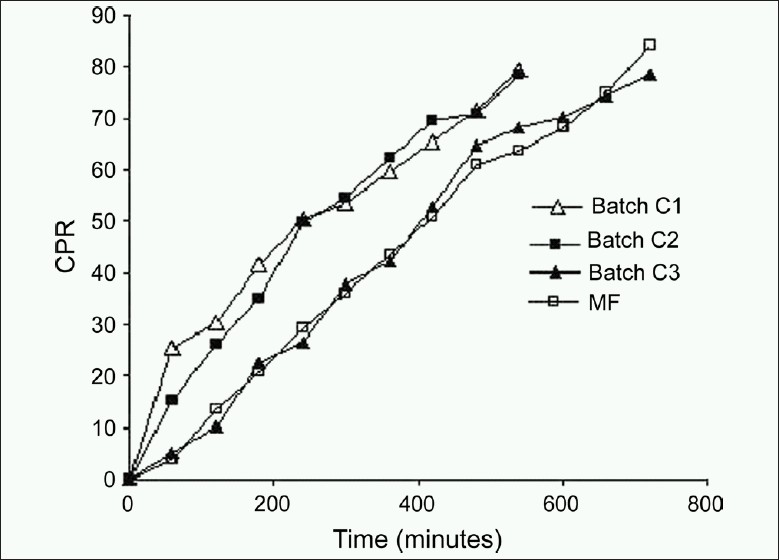
Comparative *in vitro* release profile

An ideal modified formulation is one from which a loading dose (< 30%) is released in the first hour and the remaining 70% of the drug is released thereafter at a constant rate (6.36% per hour). The following constraints were chosen for the selection of the best batch; *Y*_60_ < 30%, 52% < *Y*300 < 62% and 70% < *Y*480 < 80%. Not a single batch from F1 to F9 met all the three selection criteria. But for microspheres, drug content is an important parameter because it determines the quantity of microspheres required to deliver the dose of a drug. The microspheres of batch F2 exhibited higher drug entrapment efficiency (79.20% w/v). The microspheres showed small, discrete, good spherical geometry as shown in Figures 5 and 6. To lower the loading dose and obtain desired release profile, batch F2 microspheres were coated with enteric polymer ethylcellulose. Batch C_3_ microspheres showed the desired *in vitro* release profile compared to that of batch C1, C2, and market formulation [Figure 7]. It means that the batch C3 product mimic the release profile of DH of market formulation.

### Stability studies

Stability studies reveled that polymers used were stable and compatible with the drug and no significant difference were found in physical appearance, particle size and shape, drug content, and *in vitro* drug release during stability study, and similarity factor *f*_2_ and dissimilarity factor *f*_1_ were found to be 0.96 and 0.15, respectively.

## CONCLUSION

From the present investigation it was concluded that ethylcellulose-coated egg albumin microsphere, can be considered as an effectively coated carrier for the design of a controlled drug delivery system of highly water-soluble DH.
